# Moderate Intake of Lean Red Meat Was Associated with Lower Risk of Elevated Blood Pressure in Chinese Women: Results from the China Health and Nutrition Survey, 1991–2015

**DOI:** 10.3390/nu12051369

**Published:** 2020-05-11

**Authors:** Zhihong Wang, Qiumin Huang, Liusen Wang, Hongru Jiang, Yun Wang, Huijun Wang, Jiguo Zhang, Fengying Zhai, Bing Zhang

**Affiliations:** National Institute for Nutrition and Health, Chinese Center for Disease Control and Prevention, 29 Nanwei Road, Beijing 100050, China; wangzh@ninh.chinacdc.cn (Z.W.); qmhuangx@163.com (Q.H.); wangls@ninh.chinacdc.cn (L.W.); jianghr@ninh.chinacdc.cn (H.J.); wangyun@ninh.chinacdc.cn (Y.W.); wanghj@ninh.chinacdc.cn (H.W.); zhangjg@ninh.chinacdc.cn (J.Z.); zfy312@vip.126.com (F.Z.)

**Keywords:** fresh red meat, blood pressure, longitudinal study, China

## Abstract

This study aimed to examine longitudinal associations between fatty and lean, fresh red meat intake and blood pressure (BP) in Chinese adults. The data were from nine waves of the China Health and Nutrition Survey (1991–2015), a longitudinal, open cohort study. The surveys were conducted in 303 urban and rural communities of 15 provinces in China. Collected by consecutive three-day 24-h dietary recalls combined with household weighing for foods or only condiments, the diet exposure of interest was daily red meat intake and its subtypes (fatty versus lean) defined by 10-g fat content per 100 g. The main outcome was systolic blood pressure (SBP), diastolic blood pressure (DBP) and risk of elevated BP defined as having a mean of SBP ≥ 135 mmHg, DBP ≥ 85 mmHg, or taking antihypertensive medication. Three-level mixed-effect regressions showed women had SBP increases of 2.19 mmHg (95% CI: 1.07, 4.46) from a higher intake of total fresh red meat, 2.42 mmHg (95% CI: 1.18, 4.94) from a higher intake of fatty, fresh red meat, as well as 0.48 mmHg (95% CI: 0.26, 0.88) from a higher intake of lean, fresh red meat in the top tertile versus bottom one when adjusted for potential confounders. After adjusting for survey years, women with the highest tertile of lean, fresh red meat intake had a 32% lower risk of elevated BP (OR 0.68, 95%CI:0.48, 0.96) as compared with those with the first tertile (non-consumer). Fatty and lean, fresh red meat intakes were differentially associated with BP among Chinese adults. Further research is required to elicit the potential mechanism on gender-specific differential association of fatty versus lean, fresh red meat with BP.

## 1. Introduction

Cardiovascular diseases (CVDs) are the number one cause of death globally and China has experienced rapid increases in CVDs incidence, representing about 41% of all deaths in 2011 [1,2]. Previous studies have demonstrated the expanding burden of cardiometabolic risk factors, including elevated blood pressure (BP), in the Chinese population [1,3,4,5]. The prevalence of hypertension has been more than doubled over the past two decades [6]. It is therefore of public health importance to identify modifiable behavioral risk factors, such as unhealthy diet, to curb the hypertension epidemic and related disease burden [1].

Red meat intake has been of public health concern given that its nutrient profile rich in dietary cholesterol, saturated fat, and total fat, which have been linked to increased risk of hypertension [7,8,9]. Several cohort studies suggest an adverse association between red meat intake and BP [8] and risk of hypertension in Western populations [7,10,11,12]. Recently, some experts suggest that fatty meat, not the color of meat, is relevant to disease risk [7,10]. Potentially different relations of fatty and lean meat with BP deserve further studies. A recent Dietary Approaches to Stop Hypertension (DASH) trial shows stronger BP-lowering effect for a low-sodium DASH-type diet including 810g lean, red meat each week in US postmenstrual women aged 45 to 75 compared with similar DASH-type diet restricting red meat by the original DASH trial [10,11]. These findings demonstrate the BP-lowering effect of a combination diet, however, to date observational studies differentiating independent effects of fatty and lean, fresh red meat on BP are lacking. Wang et al. found a positive association of fatty, fresh red meat, but a null association of lean, fresh red meat, with abdominal obesity in Chinese adults [12], but whether similar associations link to BP in Chinese adults has not been previously examined.

The present study investigated the association between intake of fatty versus lean, fresh red meat and BP and elevated BP risk in Chinese adults aged 18 to 65 from the China Health and Nutrition Survey (CHNS), a longitudinal, prospective cohort study (1991–2015).

## 2. Subjects and Methods

### 2.1. Study Population

All data used in this study were derived from the CHNS. The CHNS was initiated in 1989 and has been followed up every two to four years with a focus on assessing the relationships between the economic, sociological, and demographic transformation in China and the resulting effects on the health and nutritional status of the Chinese population. The CHNS used a multistage, random cluster process to draw the sample from the original nine provinces, and communities were selected randomly as the primary sampling units. The sampling procedure has been described in detail elsewhere [13,14].

Our analysis used the nine waves of survey data between 1991 and 2015 because the CHNS collected the relevant data only from the adults aged 20–45 years in 1989. Of all the participants aged 18 to 65 who participated at least two waves of survey and had complete data on BP, diet intake, anthropometry, socio-demography, and other lifestyle factors in a survey year, we excluded pregnant or lactating women (*n* = 1016 responses), those having implausible energy intakes [15] (*n* = 307 responses; <800 kilocalories (kcal) per day or >6000 kcal for men and <600 kcal or >4000 kcal for women) and those having baseline high BP [16] (*n* = 2145 subjects; systolic blood pressure (SBP) ≥ 135 mmHg, a mean of diastolic blood pressure (DBP) ≥ 85 mmHg, or taking antihypertensive medication). The current analysis therefore consists of 8700 participants (4387 males; 4313 females) clustered in 303 communities, resulting in 17,400 total responses in the nine survey years.

The protocol of the survey was approved by the institutional review committees of the University of North Carolina at Chapel Hill and the National Institute of Nutrition and Food Safety, Chinese Center for Disease Control and Prevention. All subjects gave written informed consent for their participation in the survey.

### 2.2. Assessment of Fresh Red Meat, Fatty and Lean, Fresh Red Meat Intake

Dietary intake in the CHNS was assessed using three consecutive 24-h recalls for each individual and weighed seasonings in the household inventory over the same period. Trained health workers interviewed the participants on each of those days to collect all food consumption (type, amounts, type of meal, and place of consumption) at home and away from home during the preceding 24 hours. For the food inventory, all available purchased and stored foods in the household were measured. Changes in household food inventory as well as wastage were used to estimate total household food consumption. We determined the percentage of the oil, salt, and other condiments from the household inventory that each member consumed by the ratio of their energy intake to the energy intake of all members. The detailed diet data have been described elsewhere [13,17,18]. Based on the dietary intake of each ingredient consumed during the three days, we used the China Food Composition Table to estimate the three-day average of total energy and nutrient.

We defined fresh red meat as pork, beef, and mutton that had not been processed. We further divided fresh red meat into fatty (≥10 g fat/100 g) and lean (<10 g fat/100 g) fresh red meat based on the food grouping system developed by Popkin et al. [12,18].

### 2.3. Measurement of Blood Pressure

In each exam, the participants were in a seated position after at least five minutes of rest and with the bladder emptied in a quiet room. The trained health workers or nurses used calibrated mercury sphygmomanometers and followed a standardized procedure to measure BP of participants on their right arm. Three measurements were obtained with a 30-s interval. SBPs were determined at the first appearance of a pulse sound (Korotkoff phase 1) and DBP at the disappearance of the pulse sound (Korotkoff phase 5). We defined elevated BP as having a mean of SBP ≥ 135 mmHg, a mean of DBP ≥ 85 mmHg, or taking antihypertensive medication [19].

### 2.4. Assessment of Covariates

Trained interviewers used standard questionnaires to collect information on baseline age (in years), per capita annual family income (tertiles), individual education level (number of formally educated years), physical activity level (in MET hours/week), current smoking status (current smokers vs. former or never smokers), alcohol drinking (yes/no) and community urbanization index (score). Physical activity includes four domains: occupational, household chore, leisure time, and transportation activities. We classified the smoking status as current or ever/never. We categorized alcohol drinking status as never drinking and drinking. Participants reported all activities in average hours per week, and we converted the time spent in each activity into a metabolic equivalent of task (MET) hours per week based on the Compendium of Physical Activities [20]. The standardized, validated urbanization measure captures changes in the following 12 dimensions at the community level: population density, economic activity, traditional markets, modern markets, transportation infrastructure, sanitation, communications, housing, education, diversity, health infrastructure, and social services. Each is based on numerous measures applicable to each dimension [21]. Weight and height were measured to the nearest 0.1 kg and 0.1 cm, respectively, with the participants in lightweight clothing and without shoes. Body mass index (BMI) was calculated from measured weight (kg) and height (m) squared (kg/m2) [22]. In addition, we also assessed the intakes of other dietary factors as potential confounders, including intakes of fruit and vegetables [22], sodium [6], potassium [6], soybean and nuts, poultry and fish, and total energy intake (TEI).

### 2.5. Statistical Analysis

We first performed statistical interaction tests between dietary intakes of fresh red meat, fatty or lean, fresh red meat and gender, age, and BMI, and found the significant interaction between dietary intakes of fresh red meat and gender. We categorize the three-day average intakes of fresh red meat, fatty or lean, fresh red meat into three levels (tertiles of intake) by gender, respectively. We summarized baseline characteristics of participants and used chi-square tests for categorical variables and general linear models for continuous variables to test differences between groups and trends.

Given the hierarchical data structure of the CHNS: measurement occasions (level 1) for individuals (level 2) nested in communities (level 3), we performed three-level mixed-effects linear regression models to estimate the effect of the intakes of fresh red meat, fatty or lean, fresh red meat on SBP and DBP, respectively; and performed three-level mixed-effects logistic regression models to examine the association between intakes of fresh red meat, fatty or lean, fresh red meat and elevated BP risk. We constructed four sequential models. Model 1 includes survey years; Model 2 is additionally adjusted baseline age, income, education, urbanicity index, physical activity, and doctor-diagnosed cardiovascular disease history; Model 3 is further adjusted intakes of TEI, sodium, and other related dietary factors; Model 4 is additional further adjusted baseline BMI and baseline BP for fresh red meat, fatty, fresh red meat and lean, fresh red meat analysis, and other potential dietary confounders. In addition, we also evaluated linear trends across increasing categories of intakes of fresh red meat, fatty and lean, fresh red meat by assigning participants the median values to levels of their intake and modeled this variable as a continuous term. We used likelihood ratio tests to examine the potential confounders, effect modifier, and goodness of fit between the models.

We conducted all statistical analyses using SAS version 9.4 (SAS Institute, Inc., Cary, NC, USA) and Stata version 12.0 (StataCorp., College Station, TX, USA). All statistical tests were two-tailed and considered significant at *p* < 0.05.

## 3. Results

### 3.1. Baseline Characteristics

The selected characteristics of participants across total fresh red meat intake levels at baseline are summarized in Table 1. Men tended to have a slightly higher median intake of total fresh red meat than did women (*p* < 0.001). Both men and women with higher fresh red meat intakes tended to live in highly urbanized communities and have higher socioeconomic statuses, lower physical activity levels, and greater intakes of and total energy and other types of animal foods. It is notable that both men and women who consumed the bottle tertile of total fresh red meat had the highest intakes of soybeans and nuts and sodium and the lowest intakes of other meat across the total fresh red meat intake levels.

### 3.2. Associations of Dietary Fresh Red Meat and Its Subtypes with SBP and DBP in Participants

Table 2 and Table 3 show the longitudinal association of fresh red meat and fat-specific subtypes intake with SBP and DBP, respectively, in Chinese adults stratified by gender. After adjusting for potential confounders, women had significant SBP increase of 2.19 mmHg (95% CI: 1.07, 4.46) from a higher intake of total fresh red meat, 2.42 mmHg (95% CI: 1.18, 4.94) from a higher intake of fatty, fresh red meat, as well as 0.48 mmHg (95% CI: 0.26, 0.88) from a higher intake of lean, fresh red meat in the top tertile versus bottom one (*p*-trend < 0.05). No significant association was observed between intakes of fresh red meat and its subtypes and SBP and DBP in men. Given its linear association, women had SBP increase of 1.48 mm Hg (95% CI: 1.04, 2.11) with an increase in 50 g of total fresh red meat intake, 1.56 mm Hg (95% CI: 1.09, 2.23) with an increase in 50 g of fatty, fresh red meat intake, and 0.70 mm Hg (95% CI: 0.52, 0.95) with an increase in 50 g of lean, fresh red meat (data not shown). 

### 3.3. Associations of Dietary Fresh Red Meat and Its Subtypes with Elevated BP in Participants

Table 4 shows the odds ratio (95% CI) between levels of fresh, red meat and fat-specific subtypes consumption and elevated BP in Chinese adults stratified by gender. After adjusting for survey years, women with the highest tertile of lean, fresh red meat intake had a 32% lower risk of elevated BP (OR 0.68, 95% CI: 0.48, 0.96) as compared with those with the first tertile (non-consumer). In men, we did not find a similar association with risk of elevated BP in the comparison of the highest tertile of intake of lean, fresh red meat and non-consumers. No significant association between the intake of total fresh red meat and fatty, fresh red meat and the risk of elevated BP was observed in either men or women. In addition, women showed 17% lower risk of elevated BP (OR: 0.83, 95% CI: 0.70, 0.98; *p*-trends = 0.027) with an increase in 50 g of lean, fresh red meat but attenuated to insignificance after additional adjustment for baseline BMI, SBP, and DBP (data not shown). 

## 4. Discussion

In this large longitudinal prospective cohort study, we found that fresh red meat and fat-specific subtypes intake was positively associated with SBP in Chinese women aged 18 to 65, whereas a similar association was not observed in Chinese men. Moreover, a moderate amount of lean, fresh red meat intake was favorably associated with lower elevated BP risk compared with no intake in Chinese women.

The majority of epidemiological studies have suggested adverse effects of red meat intake on BP, elevated BP, and hypertension [23]. Several studies reported favorable effects of less red meat intake as a part of an overall dietary pattern [11,24]. Wang et al. further differentiated the relations of processed and fresh/unprocessed red meat in hypertension risk [7]. To date, studies of possibly differential roles of fatty and lean meat are limited. In China, processed red meat may not be of concern for disease risk given its very low consumption rate (<10%) and low proportion of total meat in China (about 3.1%) [25]. Our study, therefore, mainly focused on the effects of fresh red meat, fatty and lean, fresh red meat, on BP.

Of note, the definition of lean red meat varies across studies. The US MyPyramid Equivalents Database defines cooked lean meat as no more than 9.28g fat per 100 g of red meat [26]; Nowson et al. defined lean red meat as beef, lamb, veal, or combination in DASH-type diet trail [10]; Bradlee et al. defined lean red meat as beef, lamb, pork, veal and venison in which the proportion of lean part is equal or higher than 85% [27]. Our study differentiates fatty and lean, fresh red meat using 10 g of total fat per 100 g of raw pork, beef, and lamb. Previous study using this definition showed that the majority of fresh red meat consumed was fatty, fresh red meat among Chinese meat consumers (four times the intake of lean, fresh red meat), and fatty fresh pork constituted 80.0% of fresh red meat intake, almost a single source of fatty, fresh red meat intake (98.7%) in 2011 [25]. It is therefore most relevant to differentiate the effects of fatty from lean, fresh red meat due to Chinese red meat consumption patterns.

Several short-term dietary intervention studies focus on the effect of lean red meat on BP as a part of an overall dietary pattern. Nowson et al. in a recent DASH diet trial found greater, but not statistically significant, BP-lowering effect of a 14-week low-sodium DASH type diet including of an average of 85 g/day lean red meat as compared with the original DASH diet restricting red meat (SBP: 5.4 mm Hg vs. 2.7 mm Hg; DBP: 4.1 mm Hg vs. 2.9 mm Hg) in 95 postmenopausal women aged 45 to 75 [10]. Hunt et al. suggested no change in BP of 21 healthy premenopausal women who consumed a 184g meat/day-containing diet for eight weeks [28]. Hodgson and colleagues reported that energy intake from animal protein intake from energy-specific lean red meat (180 g or 250 g raw weight), as modest substitution of carbohydrate intake, may result in a 4 mm Hg reduction in BP in Australian hypertensive volunteer aged 20 and older in an eight-week parallel-design study [29].

Few observational studies have been conducted in this field. Our results showed that the intake of fresh red meat and its subtypes related slightly differently and independently to BP in Chinese men and women. The effect of the intakes of fresh red meat and fat-specific subtypes on BP was slightly different within limited amounts, SBP increase of 2.19 mmHg (95% CI: 1.07, 4.46) from a higher intake of total fresh red meat, 2.42 mmHg (95% CI: 1.18, 4.94) from a higher intake of fatty, fresh red meat, as well as 0.48 mmHg (95% CI: 0.26, 0.88) from a higher intake of lean, fresh red meat in the top tertile versus bottom one were observed in women, while the similar associations were not observed in men. In addition, lean, fresh red meat intake was only significantly linked to lower risk of elevated BP in women, whereas additional adjustment for confounders attenuated this effect to insignificance. The gender-disparity associations between BP and types of dietary fresh red meat observed in our study are possible due to the differences in physiological status and lifestyle, such as alcohol consumption and tobacco smoking, besides both the consumption of red meat and its subtypes [12] and the prevalence of hypertension [30] exist gender differences. Many factors make the comparison of our results with those of the above trials less reasonable, such as differences in study design, study population, and definition and amount of lean red meat, combined vs. independent effect trails, and short-term effect vs. longitudinal association. More prospective studies are required to confirm our findings.

The mechanisms through which the intake of lean, fresh red meat and fatty, fresh red meat differently influence BP have not been well understood. It likely reflects the combined effect of the different nutrient profiles of fatty versus lean, fresh red meat. Lean red meat is relatively lower in saturated fat (less than 1.5 g/100 g vs. more than 37 g/100 g), and higher in polyunsaturated fatty acids (PUFA) compared with visible fat from red meat, and also a good source of animal protein, mega-3 fatty acids, vitamin B12, niacin, zinc, and iron [31,32]. Saturated fat was positively associated, but PUFA was negatively associated, with the risk of increased BP and hypertension [7]. Several trials have suggested that animal protein intake from lean red meat, as modest substitution of carbohydrate intake, may lower BP in hypertensive persons due to relative low dietary acid load [10,29,33], whereas population studies have shown a null association of animal protein with BP and risk of hypertension [34]. One study showed that heme iron intake was positively but not significantly associated with BP [35]. Overall, the mechanism of the favorable role of lean, fresh red meat intake within reasonable limits in lowering BP needs further investigation.

To the best of our knowledge, our study is the first to investigate the differential association of fatty versus lean, fresh red meat with BP and the risk of elevated BP in the Chinese population. The 20-year longitudinal, prospective study design, the standardized procedure for measuring SBP and DBP three times during each exam, and comprehensive adjustment for potential confounders are all strengths of this study. Moreover, individual fresh red meat intake was assessed every two to four years and the examination of the longitudinal association of fresh red meat intake with BP are important given probably similar time frame-related effect magnitude as sodium intake, i.e., a stronger association of the most recent exposure with hypertension risk compared with baseline and cumulative exposure [36]. Further, our study provides a more precise effect estimate given many advantages of multilevel mixed-effect modeling instead of traditional regression analyses [26,37,38].

Several potential limitations of our study deserve discussion. First, 24 h recall has a relatively limited ability to assess usual dietary intake, especially for episodically consumed red meat in rural adults in the earlier waves of surveys. However, the average intake of three 24 h dietary recall can reduce measurement error and provide a relatively precise estimate [39,40]. Second, we cannot exclude the possibility of residual confounding given the nature of observational studies. Third, given that the CHNS did not collect enough information to identify treatments with cortisone, contraceptives or others, we cannot evaluate such treatments, which may underestimate the level of SBP or DBP, and the prevalence odds of elevated BP in relation to the dietary fresh red meat intakes. Fourth, the mean age values at baseline were about 40 years (about 25% of baseline subjects were over 50 years old in our study, and about 10% were over 60 years old) and increased in the following longitudinal survey. Given the elderly may change their diet intakes due to cardiometabolic risk factors or chronic diseases and misestimated the effect magnitude, we selected the adults aged 18 to 65 as the subjects, and took accounting the correlation between age and fresh red meat intake, as well as age as a risk factor of BP and elevated BP. We therefore regarded ‘age’ as a confounding factor to justify the potential bias and kept adjusting the baseline age instead of dynamic age as one confounder. Finally, 10 g fat/100 g fresh red meat cutoff differentiating fatty and lean, fresh red meat in our study is based on total fat and relatively arbitrary. Further, composition and amount of fatty acid consumption may play a powerful role in disease risk [41,42]. Future studies are needed to add further evidence on a more meaningful and evidence-based fatty acid-related definition of fatty and lean meat.

## 5. Conclusions

In conclusion, the present study suggested that fatty and lean, fresh red meat intakes were differentially associated with BP among Chinese adults and provided some evidence of a favorable role of lean, fresh red meat in lowering BP. It is of public health relevance that a moderate intake of lean, fresh red meat was protective for elevated BP risk in women, which was consistent with the current findings of lean red meat from DASH related studies. Chinese dietary guideline recommends moderate intakes of fish, poultry, egg, and lean red meat. The potential gender-specific mechanism and clinic meaning of intakes of fatty and lean, fresh red meat in relation to BP warrant further studies.

## Figures and Tables

**Table 1:** Baseline characteristics of participants according to three levels of total fresh red meat consumption by gender, CHNS.

Variables	Men			*p*	Women			*p*
	T1 (*n* = 1434)	T2 (*n* = 1491)	T3 (*n* = 1462)		T1 (*n* = 1392)	T2 (*n* = 1490)	T3 (*n* = 1431)	
Fresh red meat median intake (g/day) ^1^	33.33 (21.43, 50.00)	83.33 (66.67, 100.00)	166.67 (134.62, 208.33)		33.33 (16.67, 34.62)	66.67 (55.00, 83.33)	133.33 (110.00, 166.67)	<0.001
Age (years) ^2^	37.26 ± 0.30	36.13 ± 0.30	35.91 ± 0.30	0.003	38.86 ± 0.30	38.49 ± 0.29	37.71 ± 0.29	0.020
Income level (%)								
Low	40.24	32.19	27.56	<0.001	41.02	32.08	27.04	<0.001
Medium	31.66	33.67	34.82		32.18	33.56	34.38	
High	28.10	34.14	37.62		26.80	34.36	38.57	
Education (years)	8.60 ± 0.09	9.03 ± 0.09	9.48 ± 0.09	<0.001	7.18 ± 0.11	7.65 ± 0.11	8.42 ± 0.11	<0.001
Urbanicity index	55.43 ± 0.52	60.61 ± 0.51	64.48 ± 0.52	<0.001	56.79 ± 0.53	61.22 ± 0.51	67.05 ± 0.52	<0.001
Physical activity (MET hours/week)	323.86 ± 5.87	267.52 ± 5.75	238.62 ± 5.81	<0.001	343.37 ± 6.59	294.98 ± 6.37	257.66 ± 6.50	<0.001
Current smokers (%)	62.83	59.96	64.16	0.055	3.95	2.55	2.73	0.062
Alcohol drinking (%)	61.58	62.58	64.36	0.289	12.50	11.07	13.07	0.236
BMI (kg/m^2^)	22.12 ± 0.08	22.05 ± 0.08	22.19 ± 0.08	0.472	22.24 ± 0.08	22.36 ± 0.08	22.19 ± 0.08	0.332
SBP(mmHg)	113.22 ± 0.27	113.60 ± 0.26	113.73 ± 0.26	0.370	109.26 ± 0.29	109.15 ± 0.28	109.72 ± 0.29	0.331
DBP (mmHg)	73.62 ± 0.19	73.74 ± 0.19	73.93 ± 0.19	0.508	71.52 ± 0.21	71.49 ± 0.20	71.70 ± 0.20	0.727
Dietary intake								
Total energy (kcal/day)	2485.02 ± 19.69	2635.24 ± 19.29	2961.98 ± 19.49	<0.001	2076.69 ± 16.12	2216.17 ± 15.58	2410.26 ± 15.91	<0.001
Fatty, fresh red meat (g/day)	30.73 ± 1.22	68.04 ± 1.18	133.19 ± 1.22	<0.001	24.51 ± 1.03	53.68 ± 0.99	107.71 ± 1.02	<0.001
Lean, fresh red meat (g/day)	4.80 ± 1.14	16.90 ± 1.10	48.11 ± 1.14	<0.001	4.54 ± 0.88	14.35 ± 0.85	38.64 ± 0.88	<0.001
Poultry (g/day)	12.59 ± 1.06	15.91 ± 1.03	21.48 ± 1.06	<0.001	9.82 ± 0.93	13.10 ± 0.89	16.46 ± 0.92	<0.001
Fish (g/day)	28.19 ± 1.52	34.35 ± 1.48	41.22 ± 1.52	<0.001	24.49 ± 1.34	29.37 ± 1.28	36.07 ± 1.33	<0.001
Vegetables and fruits (g/day)	388.01 ± 5.36	386.55 ± 5.19	385.03 ± 5.35	0.928	362.34 ± 5.15	366.60 ± 4.92	372.90 ± 5.09	0.349
Soybeans and nuts (g/day)	30.93 ± 1.00	26.38 ± 0.97	22.61 ± 1.00	<0.001	26.89 ± 0.95	25.49 ± 0.91	21.33 ± 0.94	<0.001
Sodium (mg/day)	6838.14 ± 116.37	6308.54 ± 112.71	6474.76 ± 116.13	0.004	5859.05 ± 98.30	5728.98 ± 102.86	5444.01 ± 101.74	0.012
Potassium (mg/day)	1281.89 ± 8.19	1262.06 ± 7.94	1271.70 ± 8.18	0.219	1090.90 ± 6.91	1065.77 ± 6.60	1105.13 ± 6.83	<0.001

T = tertile; SBP = systolic blood pressure; DBP = diastolic blood pressure; MET = metabolic equivalent. ^1^ Median; interquartile range in parentheses. ^2^ Mean ± standard error (all such values). Chi-square tests for categorical variables and general linear models for continuous variables tested differences between groups and trends. Adjusted by age for physical activity, total energy intake, BMI, SBP, and DBP and adjusted by age and total energy intake for other food groups and nutrients.

**Table 2:** Regression coefficients (95% CI) of SBP and DBP according to level of intake of total fresh red meat and subtypes among male Chinese, CHNS ^1^.

Variables	SBP			*p*-trend ^2^	DBP			*p*-trend ^2^
	T1	T2	T3		T1	T2	T3	
Total fresh red meat								
Model 1 ^3^	Reference	1.24 (0.65, 2.38)	1.45 (0.73, 2.85)	0.302	Reference	1.45 (0.73, 2.85)	1.28 (0.80, 2.04)	0.253
Model 2	Reference	1.40 (0.74, 2.62)	1.65 (0.85, 3.20)	0.148	Reference	1.65 (0.85, 3.20)	1.26 (0.80, 1.98)	0.261
Model 3	Reference	1.27 (0.67, 2.40)	1.23 (0.61, 2.48)	0.598	Reference	1.23 (0.61, 2.48)	1.16 (0.72, 1.88)	0.463
Model 4 ^4^	Reference	1.04 (0.60, 1.83)	0.88 (0.48, 1.63)	0.689	Reference	0.88 (0.48, 1.63)	0.89 (0.58, 1.35)	0.654
Fatty, fresh red meat								
Model 1 ^3^	Reference	0.62 (0.32, 1.18)	1.03 (0.53, 1.99)	0.813	Reference	0.62 (0.40, 1.01)	0.97 (0.62, 1.53)	0.898
Model 2	Reference	0.71 (0.38, 1.33)	1.18 (0.62, 2.24)	0.518	Reference	0.68 (0.44, 1.04)	1.01 (0.65, 1.57)	0.781
Model 3	Reference	0.67 (0.35, 1.25)	0.92 (0.47, 1.81)	0.880	Reference	0.68 (0.44, 1.05)	0.97 (0.61, 1.54)	0.973
Model 4 ^4^	Reference	0.89 (0.51, 1.56)	0.82 (0.45, 1.49)	0.499	Reference	0.94 (0.64, 1.38)	0.81 (0.54, 1.23)	0.279
Lean, fresh red meat								
Model 1 ^3^	Reference	0.65 (0.27, 1.56)	1.33 (0.70, 2.53)	0.415	Reference	1.13 (0.62, 2.08)	1.16 (0.75, 1.81)	0.567
Model 2	Reference	0.69 (0.29, 1.62)	1.36 (0.73, 2.54)	0.375	Reference	1.06 (0.59, 1.92)	1.05 (0.69, 1.62)	0.894
Model 3	Reference	0.72 (0.31, 1.70)	1.23 (0.65, 2.33)	0.564	Reference	1.06 (0.59, 1.92)	0.98 (0.63, 1.51)	0.827
Model 4 ^4^	Reference	0.67 (0.32, 1.44)	0.94 (0.54, 1.65)	0.786	Reference	1.00 (0.60, 1.69)	0.91 (0.62, 1.33)	0.571

T = tertile; SBP = systolic blood pressure; DBP=diastolic blood pressure. ^1^ All of the models were performed three-level mixed-effects linear regression with maximum likelihood estimation methods and the bottom tertile of each type of fresh red meat as the reference group. ^2^ *p*-trend was calculated across the tertiles of each type of fresh red meat among consumers, and this variable was entered as a continuous term in the regression models. ^3^ Model 1 adjusted for survey years only; model 2 additionally adjusted for baseline age, individual income, education level, urbanicity index, physical activity, and disease history; model 3 further adjusted for total energy intake, and intakes of other food groups. ^4^ model 4 plus baseline BMI and baseline SBP and DBP for total fresh red meat, fatty, fresh red meat and lean, fresh red meat analysis; lean, fresh red meat only for fatty, fresh red meat analysis or fatty, fresh red meat only for lean, fresh red meat analysis.

**Table 3:** Regression coefficients (95% CI) of SBP and DBP according to level of intake of total fresh red meat and subtypes among female Chinese, CHNS ^1^.

Variables	SBP			*p*-trend ^2^	DBP			*p*-trend ^2^
	T1	T2	T3		T1	T2	T3	
Total fresh red meat								
Model 1 ^3^	Reference	1.59 (0.79, 3.22)	1.62 (0.78, 3.39)	0.225	Reference	1.15 (0.73, 1.81)	1.21 (0.75, 1.94)	0.348
Model 2	Reference	1.55 (0.79, 3.04)	2.19 (1.07, 4.46) *	0.027	Reference	1.10 (0.71, 1.71)	1.31 (0.82, 2.07)	0.164
Model 3	Reference	1.51 (0.77, 2.99)	1.93 (0.91, 4.09)	0.079	Reference	1.09 (0.70, 1.69)	1.22 (0.75, 1.98)	0.297
Model 4 ^4^	Reference	1.50 (0.82, 2.74)	1.54 (0.80, 2.97)	0.186	Reference	1.14 (0.78, 1.65)	1.16 (0.77, 1.76)	0.338
Fatty, fresh red meat								
Model 1 ^3^	Reference	1.64 (0.82, 3.29)	2.22 (1.10, 4.50) *	0.028	Reference	1.21 (0.78, 1.90)	1.21 (0.78, 1.86)	0.189
Model 2	Reference	1.68 (0.86, 3.26)	2.52 (1.28, 4.95) **	0.008	Reference	1.36 (0.87, 2.15)	1.41 (0.91, 2.18)	0.135
Model 3	Reference	1.71 (0.88, 3.34)	2.42 (1.18, 4.94) *	0.017	Reference	0.73 (0.38, 1.41)	0.79 (0.42, 1.48)	0.176
Model 4 ^4^	Reference	1.35 (0.74, 2.46)	1.86 (0.98, 3.52)	0.049	Reference	0.63 (0.40, 1.01)	0.69 (0.45, 1.07)	0.213
Lean, fresh red meat								
Model 1 ^3^	Reference	0.40 (0.15, 1.12)	0.26 (0.13, 0.53) ***	0.001	Reference	1.24 (0.80, 1.91)	1.21 (0.83, 1.76)	0.059
Model 2	Reference	0.50 (0.19, 1.34)	0.35 (0.18, 0.68) **	0.006	Reference	1.40 (0.88, 2.22)	1.30 (0.87, 1.94)	0.122
Model 3	Reference	0.52 (0.20, 1.39)	0.32 (0.16, 0.63) ***	0.003	Reference	0.79 (0.42, 1.49)	1.04 (0.60, 1.79)	0.063
Model 4 ^4^	Reference	0.97 (0.41, 2.31)	0.48 (0.26, 0.88) *	0.040	Reference	0.65 (0.42, 1.00)	0.74 (0.51, 1.08)	0.153

T = tertile; SBP = systolic blood pressure; DBP = diastolic blood pressure. ^1^ All of the models were performed three-level mixed-effects linear regression with maximum likelihood estimation methods and the bottom tertile of each type of fresh red meat as the reference group. ^2^ *p*-trend was calculated across the tertiles of each type of fresh red meat among consumers, and this variable was entered as a continuous term in the regression models. ^3^ Model 1 adjusted for survey years only; model 2 additionally adjusted for baseline age, individual income, education level, urbanicity index, physical activity, and disease history; model 3 further adjusted for total energy intake, and intakes of other food groups. ^4^ model 4 plus baseline BMI and baseline SBP and DBP for total fresh red meat, fatty, fresh red meat and lean, fresh red meat analysis; lean, fresh red meat only for fatty, fresh red meat analysis or fatty, fresh red meat only for lean, fresh red meat analysis. * *p* < 0.050, ** *p* < 0.010, *** *p* < 0.001.

**Table 4:** Odds ratio (95% CI) of elevated BP according to three levels of intake of total fresh red meat and its subtypes among Chinese adults, CHNS ^1.^

	Elevated BP (Men)			*p*-trend ^3^	Elevated BP (Women)			*p*-trend ^3^
	T1	T2	T3		T1	T2	T3	
Total fresh red meat intake (g/day) ^2^	33.3 (21.4, 50.0)	83.3 (66.7, 100.0)	166.7 (134.6, 208.3)		33.3 (16.7, 34.6)	66.7 (55.0, 83.3)	133.3 (110.0, 166.7)	
Model 1 ^4^	Reference	0.91 (0.76, 1.10)	0.98 (0.81, 1.19)	0.942	Reference	1.00 (0.68, 1.48)	0.85 (0.55, 1.30)	0.530
Model 2	Reference	0.95 (0.79, 1.15)	1.05 (0.87, 1.28)	0.535	Reference	1.12 (0.90, 1.40)	1.05 (0.83, 1.33)	0.667
Model 3	Reference	0.94 (0.77, 1.13)	0.99 (0.81, 1.22)	0.991	Reference	1.12 (0.89, 1.40)	1.05 (0.81, 1.35)	0.676
Model 4 ^5^	Reference	0.91 (0.77, 1.08)	0.95 (0.79, 1.15)	0.672	Reference	1.12 (0.91, 1.38)	1.02 (0.80, 1.29)	0.832
Fatty, fresh red meat intake (g/day) ^2^	16.7 (0.0, 33.3)	66.7 (50.0, 75.0)	133.3 (106.7, 166.7)		16.7 (0.0, 28.6)	50.0 (46.7, 66.7)	107.1 (89.3, 141.7)	
Model 1 ^4^	Reference	0.95 (0.79, 1.15)	1.01 (0.83, 1.22)	0.940	Reference	1 (0.67, 1.50)	1.03 (0.68, 1.56)	0.881
Model 2	Reference	1.01 (0.84, 1.21)	1.07 (0.89, 1.30)	0.490	Reference	1.06 (0.85, 1.32)	1.09 (0.86, 1.36)	0.500
Model 3	Reference	0.99 (0.82, 1.19)	1.01 (0.83, 1.24)	0.953	Reference	1.06 (0.85, 1.33)	1.10 (0.86, 1.40)	0.480
Model 4 ^5^	Reference	1.04 (0.85, 1.26)	1.1 (0.80, 1.52)	0.816	Reference	1.05 (0.85, 1.29)	1.05 (0.84, 1.32)	0.688
Lean, fresh red meat intake (g/day) ^2^	0.0 (0.0, 0.0)	16.7 (16.7, 26.7)	66.7 (50.0, 100.0)		0.0 (0.0, 0.0)	16.7 (13.3, 21.4)	57.4 (36.7, 83.3)	
Model 1 ^4^	Reference	0.96 (0.75, 1.23)	1.04 (0.87, 1.25)	0.723	Reference	0.85 (0.53, 1.35)	0.68 (0.48, 0.96) *	0.038
Model 2	Reference	1.03 (0.80, 1.32)	1.09 (0.90, 1.31)	0.449	Reference	0.92 (0.66, 1.28)	0.84 (0.67, 1.05)	0.138
Model 3	Reference	1.04 (0.81, 1.34)	1.08 (0.90, 1.31)	0.469	Reference	0.92 (0.66, 1.29)	0.83(0.66, 1.05)	0.141
Model 4 ^5^	Reference	1.08 (0.85, 1.38)	1.05 (0.80, 1.39)	0.731	Reference	0.91 (0.67, 1.24)	0.81 (0.65, 1.01)	0.076

T= tertile; BP= blood pressure. ^1^ All of the models were constructed three-level mixed-effects logistic regression and the bottom tertile of each type of fresh red meat as the reference group. ^2^ Median; interquartile range in parentheses. ^3^ *p*-trend across categories of intake of total fresh red meat and fatty or lean, fresh red meat was calculated using the Wald test by assigning to each subject the median value for his/her consumption category and modeling this term as a continuous variable. ^4^ Model 1 adjusted for survey years only; model 2 additionally adjusted for baseline age, individual income, education level, urbanicity index, physical activity, and disease history; model 3 further adjusted for total energy intake, and intakes of other food groups. ^5^ Model 4 plus baseline BMI, SBP, and DBP for fresh red meat, fatty, fresh red meat and lean, fresh red meat analysis; lean, fresh red meat only for fatty, fresh red meat analysis or fatty, fresh red meat only for lean, fresh red meat analysis. * *p* < 0.050.
